# Supplementary material for: Heterozygous expression of a Kcnt1 gain-of-function variant has differential effects on somatostatin- and parvalbumin-expressing cortical GABAergic neurons
Source: eLife. 2024 Oct 11;13:RP92915. doi: 10.7554/eLife.92915 (PMC11469685; doi:10.7554/eLife.92915)
Supplement: Supplementary file 1. — For an explanation of the parameters, see Methods. Rin = input resistance, Tau = membrane time constant, Rheo = rheobase current, Cm = membrane capacitance, AP = action potential, thresh = threshold, amp = amplitude, hw = half width, AHP = afterhyperpolarization, and mfr = maximum firing rate. Values shown are estimated marginal means ±the standard error as determined by implementing a generalized linear mixed model. p-values less than 0.05 are in red, bold type. For each subgroup, N values are the number of mouse pups, and n values are the number of neurons. [file elife-92915-supp1.docx]

**Supplementary file 1. Electrophysiological parameters of current clamp recordings from neuronal cultures.**

|  | **Glutamatergic** | | | **FS GABAergic** | | | **NFS GABAergic** | | |
| --- | --- | --- | --- | --- | --- | --- | --- | --- | --- |
|  | WT | YH-HET |  | WT | YH-HET |  | WT | YH-HET |  |
|  | N = 9 | N = 9 |  | N = 9 | N = 9 |  | N = 9 | N = 9 |  |
|  | n = 27 | n = 30 | p-value | n = 27 | n = 29 | p-value | n = 26 | n = 22 | p-value |
| V_rest_ (mV) | -57.9±1.6 | -57.0±1.7 | 0.61 | -53.8±1.8 | -52.8±1.8 | 0.60 | -56.3±1.8 | -54.4±1.9 | 0.34 |
| R_in_ (MΩ) | 177±17 | 182±16 | 0.80 | 95±9 | 105±10 | 0.40 | 171±16 | 146±14 | 0.19 |
| Tau (ms) | 32.9±3.8 | 39.3±4.3 | 0.14 | 17.3±2.0 | 16.7±1.9 | 0.75 | 24.9±2.9 | 22.2±2.7 | 0.37 |
| C_m_ (pF) | 198±15 | 215±16 | 0.23 | 183±14 | 163±12 | 0.15 | 143±11 | 164±13 | 0.09 |
| Rheo (pA) | 171±19 | 148±16 | 0.25 | 400±45 | 415±47 | 0.78 | 196±22 | 297±36 | **0.005** |
| AP_thresh_ (mV) | -27.7±1.6 | 27.1±1.4 | 0.98 | -25.7±1.6 | -25.8±1.6 | 0.95 | -28.2±1.6 | -24.4±1.7 | **0.012** |
| AP_amp_ (mV) | 77.8±2.0 | 77.5±1.5 | 0.51 | 70.9±1.8 | 67.8±1.7 | 0.15 | 71.5±1.8 | 68.7±1.9 | 0.28 |
| AP_hw_ (ms) | 2.19±0.12 | 2.20±0.12 | 0.95 | 0.89±0.05 | 0.91±0.05 | 0.85 | 1.36±0.08 | 1.11±0.07 | **0.01** |
| AHP (mV) | 11.8±0.6 | 11.8±0.6 | 0.96 | 27.5±1.3 | 26.9±1.4 | 0.78 | 19.3±1.0 | 22.6±1.2 | **0.034** |
| AP_mfr_ (Hz) | 22.4±2.3 | 22.4±2.4 | 0.99 | 68.8±6.2 | 71.4±6.6 | 0.53 | 35.0±3.8 | 31.8±3.5 | 0.25 |

For an explanation of the parameters, see Methods. R_in_ = input resistance, Tau = membrane time constant, Rheo = rheobase current, C_m_ = membrane capacitance, AP = action potential, thresh = threshold, amp = amplitude, hw = half width, AHP = afterhyperpolarization, and mfr = maximum firing rate. Values shown are estimated marginal means ± the standard error as determined by implementing a Generalized Linear Mixed Model. P values less than 0.05 are in red, bold type. For each subgroup, N values are the number of mouse pups, and n values are the number of neurons.
