# Supplementary material for: Heterozygous expression of a Kcnt1 gain-of-function variant has differential effects on somatostatin- and parvalbumin-expressing cortical GABAergic neurons
Source: eLife. 2024 Oct 11;13:RP92915. doi: 10.7554/eLife.92915 (PMC11469685; doi:10.7554/eLife.92915)
Supplement: Supplementary file 2. — Table shows values (mean ± SEM) of all electrophysiological parameters measured from WT and YH-HET, SST, VIP, and PV GABAergic neurons. For an explanation of the parameters, see Methods. Rin = input resistance, Tau = membrane time constant, Rheo = rheobase current, Cm = membrane capacitance, AP = action potential, thresh = threshold, amp = amplitude, hw = half width, AHP = afterhyperpolarization, and mfr = maximum firing rate. Values shown are estimated marginal means ± the standard error as determined by implementing a generalized linear mixed model. p values less than 0.05 are in red, bold type. For each subgroup, N values are the number of mouse pups, and n values are the number of neurons. [file elife-92915-supp2.docx]

**Supplementary file 2. Electrophysiological parameters of current clamp recordings from neuronal cultures of three major GABAergic subtypes.**

|  | **SST^+^ GABAergic** | | | **VIP^+^ GABAergic** | | | **PV^+^ GABAergic** | | |
| --- | --- | --- | --- | --- | --- | --- | --- | --- | --- |
|  | WT | YH-HET |  | WT | YH-HET |  | WT | YH-HET |  |
|  | N = 4 | N = 5 |  | N = 3 | N = 4 |  | N = 3 | N = 4 |  |
|  | n = 45 | n = 46 | p-value | n = 50 | n = 48 | p-value | n = 23 | n = 25 | p-value |
| R_in_ (MΩ) | 133±18 | 95±13 | **0.001** | 243±10 | 249±11 | 0.645 | 67±4 | 66±3 | 0.850 |
| Tau (ms) | 16.9±1.1 | 13.5±0.8 | **0.012** | 35.1±2.4 | 33.1±2.3 | 0.384 | 6.7±0.5 | 6.0±0.4 | 0.302 |
| C_m_ (pF) | 135±13 | 152±15 | **0.032** | 148±6 | 134±6 | 0.055 | 103±5 | 90±4 | 0.050 |
| Rheo (pA) | 232±41 | 319±56 | **0.001** | 149±12 | 157±13 | 0.344 | 546±33 | 441±24 | **0.031** |
| AP_thresh_ (mV) | -36.4±0.8 | -35.2±0.8 | 0.113 | -30.9±0.4 | -31.0±0.4 | 0.850 | -34.2±1.8 | -35.5±1.9 | 0.308 |
| AP_amp_ (mV) | 79.9±1.5 | 80.2±1.5 | 0.850 | 75.6±0.9 | 75.5±0.9 | 0.943 | 77.6±1.0 | 82.7±1.0 | **0.002** |
| AP_hw_ (ms) | 0.91±0.02 | 0.86±0.02 | 0.142 | 1.09±0.04 | 1.09±0.04 | 0.916 | 0.73±0.03 | 0.69±0.02 | 0.180 |
| AHP (mV) | 23.0±0.7 | 23.5±0.7 | 0.554 | 17.2±0.9 | 17.4±0.9 | 0.860 | 26.6±0.6 | 25.3±0.5 | 0.150 |
| AP_mfr_ (Hz) | 57.6±5.1 | 55.7±4.9 | 0.432 | 35.5±2.8 | 35.9±2.8 | 0.857 | 69.4±4.9 | 79.0±5.3 | 0.068 |

For an explanation of the parameters, see Methods. R_in_ = input resistance, Tau = membrane time constant, Rheo = rheobase current, C_m_ = membrane capacitance, AP = action potential, thresh = threshold, amp = amplitude, hw = half width, AHP = afterhyperpolarization, and mfr = maximum firing rate. Values shown are estimated marginal means ± the standard error as determined by implementing a Generalized Linear Mixed Model. P values less than 0.05 are in red, bold type. For each subgroup, N values are the number of mouse pups, and n values are the number of neurons.
