# Supplementary material for: Heterozygous expression of a Kcnt1 gain-of-function variant has differential effects on somatostatin- and parvalbumin-expressing cortical GABAergic neurons
Source: eLife. 2024 Oct 11;13:RP92915. doi: 10.7554/eLife.92915 (PMC11469685; doi:10.7554/eLife.92915)
Supplement: Supplementary file 3. — Table shows values (mean ± SEM) of all electrophysiological parameters measured in silico from VIP, SST, and PV GABAergic neurons with varying levels of KCNT1 GOF (and, in some cases, INaP). For an explanation of the parameters, see Methods. AP = action potential. For each subgroup, 10 model neurons were used and simulations were repeated with the EC50 (in mM) for Na+ for the KCNT1 conductance set at the indicated levels. ‘+SST’ refers to the activation curve parameters (V50 and slope) for the KCNT1 conductance in that neuron type being replaced with those measured in SST neurons. Values shown are means ± the standard error. The statistical test is a repeated measures ANOVA. [file elife-92915-supp3.docx]

| Model neuron | Input  Resistance (MΩ) | p-value  vs 40 mM | Rheobase (pA) | p-value  vs 40 mM | AP rate at 1.5x Rheobase (Hz) | p-value  vs 40 mM |
| --- | --- | --- | --- | --- | --- | --- |
| VIP-40 mM | 266.5 ± 28.2 |  | 78.7 ± 3.7 |  | 7.0 ± 0.7 |  |
| VIP-35 mM | 266.2 ± 28.1 | 0.193 | 79.1 ± 3.6 | 0.177 | 7.0 ± 0.7 | 0.999 |
| VIP-30 mM | 264.8 ± 28.0 | 0.012 | 80.4 ± 3.5 | 0.014 | 6.9 ± 0.7 | 0.527 |
| VIP-30 mM + SST | 260.2 ± 27.8 | <0.001 | 86.3 ± 3.8 | <0.001 | 6.5 ± 0.8 | 0.011 |
|  |  |  |  |  |  |  |
| SST-40 mM | 210.3 ± 23.0 |  | 130.0 ± 9.1 |  | 17.3 ± 2.1 |  |
| SST-35 mM | 199.9 ± 22.1 | 0.007 | 162.5 ± 11.9 | <0.001 | 11.9 ± 2.0 | 0.025 |
| SST-30 mM | 187.1 ± 21.2 | 0.001 | 187.0 ± 12.5 | <0.001 | 7.1 ± 1.9 | 0.003 |
|  |  |  |  |  |  |  |
| PV-40 mM | 105.8 ± 7.7 |  | 258.0 ± 7.5 |  | 18.3 ± 1.3 |  |
| PV-35 mM | 105.5 ± 7.7 | 0.140 | 264.5 ± 7.9 | 0.004 | 16.8 ± 1.4 | 0.033 |
| PV-30 mM | 103.3 ± 7.5 | <0.001 | 273.0 ± 9.5 | 0.004 | 12.4 ± 2.0 | 0.004 |
| PV-35 mM + I_NaP_ | 104.6 ± 7.5 | <0.001 | 238.5 6.3 | <0.001 | 21.1 ± 1.3 | <0.001 |
| PV-30 mM + I_NaP_ | 103.4 ± 7.4 | 0.009 | 250.5 ± 6.9 | 0.002 | 20.1 ± 1.5 | 0.012 |
|  |  |  |  |  |  |  |
| Glut-40 mM | 147.1 ± 12.0 |  | 117.0 ± 10.4 |  | 2.46 ± 0.3 |  |
| Glut-30 mM | 146.4 ± 12.1 | 0.017 | 121.5 ± 10.0 | <0.001 | 2.36 ± 0.4 | 0.593 |
| Glut-30 mM + SST | 140.4 ± 11.9 | 0.001 | 146.0 ± 9.7 | <0.001 | 1.54 ± 0.3 | 0.012 |

**Supplementary file 3. Electrophysiological parameters using compartmental model neurons.**

For an explanation of the parameters, see Methods. AP = action potential. For each subgroup, 10 model neurons were used and simulations were repeated with the EC_50_ (in mM) for Na^+^ for the KCNT1 conductance set at the indicated levels. “+ SST” refers to the activation curve parameters (V_50_ and slope) for the KCNT1 conductance in that neuron type being replaced with those measured in SST neurons. Values shown are means ± the standard error. The statistical test is a repeated measures ANOVA.
